# Hierarchical friction memory leads to subdiffusive configurational dynamics of fast-folding proteins

**DOI:** 10.1073/pnas.2516506123

**Published:** 2026-02-06

**Authors:** Anton Klimek, Benjamin A. Dalton, Lucas Tepper, Roland R. Netz

**Affiliations:** ^a^Fachbereich Physik, Freie Universität Berlin, Berlin 14195, Germany

**Keywords:** protein folding, subdiffusion, non-Markovian dynamics

## Abstract

Proteins often exhibit subdiffusive configurational dynamics, the origins of which are still unresolved. We investigate the impact of non-Markovian friction and the free-energy landscape on the dynamics of fast-folding proteins in terms of the mean squared displacement (MSD) and the mean first-passage-time (MFPT) of the folding reaction coordinate. We find the friction memory kernel from published molecular dynamics simulations to be well-described by a hierarchical multiexponential function, which gives rise to subdiffusion in the MSD for times shorter than the longest memory time, while for longer times the confining free-energy landscape produces subdiffusion. Thus, for a wide range of times, friction memory effects in fast-folding proteins dominate the scaling behavior of the MSD compared to effects due to the folding free-energy landscape. As a consequence, Markovian models are insufficient to fully capture the folding dynamics, as quantified by the MSD and the MFPT, even when including coordinate-dependent friction. Our results demonstrate the importance of memory effects in protein folding and conformational dynamics and explicitly show that subdiffusion in fast-folding protein dynamics originates mainly from memory effects, not from the free-energy landscape and not from coordinate-dependent friction.

## Significance

The dynamics of protein folding is relevant for many biological functions. Most models using a free-energy folding landscape rely on instantaneous (i.e. memoryless) friction. We find that the time-dependent friction memory extracted from molecular-dynamics simulations for a set of fast-folding proteins is well described by a hierarchical structure of exponentials decaying in time. We show that this friction memory is needed to reproduce the correct protein folding dynamics and responsible for power-law behavior of the mean squared displacement with the correct subdiffusive exponent over a finite range of time. This reconciles the common views of hierarchical protein folding and subdiffusive protein dynamics.

Almost every biological process relies on the functioning of proteins, which is strongly dependent on their specific three-dimensional arrangement. Whereas recent tools such as alpha fold allow fast and accurate predictions of the folded structure of a protein for a given amino acid sequence (1), they do not offer any information about the dynamics of the folding that leads to that conformation. However, dynamical information is crucial for many applications, such as the development of interventions of misfolding (2). Since a fully atomistic description of a protein involves many degrees of freedom, the structure is often projected on low-dimensional reaction coordinates (RC) (3, 4), or coarse-grained into subunits (5–7). The choice of a suitable RC is highly nontrivial. Usually, one assumes the RC to be Markovian (8), i.e. to exhibit no dependence on past dynamics but only on the present state, in which case a description of the dynamics in terms of static friction and the free-energy landscape of the RC is possible (9). Alternatively, one can use Markov state models to identify protein folding pathways (10, 11). However, it has recently been shown that, in the case of protein folding, the dynamics of many RCs exhibit significant non-Markovian behavior (12, 13). This suggests that finite-timescale intramolecular and solvent relaxation processes are significant and cannot be neglected. In addition, experimentally only certain RCs are measurable, which makes it practically impossible to observe a Markovian RC. Thus, there have been numerous studies that focus on non-Markovian effects in protein conformational dynamics (12–19). These studies rely on the generalized Langevin equation (GLE), an equation of motion derived from the underlying many-body Hamiltonian (20–22). For a given RC q(t), the GLE reads

[1] $$\begin{array}{c} m\ddot{q}\left(t\right)=-\nabla U\left(q\left(t\right)\right)-\int_{0}^{t}\Gamma \left(t-t^{\prime }\right)\dot{q}\left(t^{\prime }\right)dt^{\prime }+F_{R}\left(t\right), \end{array}$$

where −∇U(q) is the force due to the free-energy landscape U(q), $\dot{q}\left(t\right)$ denotes the velocity and $\ddot{q}\left(t\right)$ the acceleration of the RC, and Γ(t) is the friction memory kernel, which incorporates the non-Markovian effects. The friction kernel Γ(t) and the random force $F_{R}\left(t\right)$, as well as the free energy U(q) originate from the projection of the many-body dynamics on a single RC. In an equilibrium scenario, the mass, which is approximated here to be independent of q, is defined by an analog of the equipartition theorem as $m=k_{B}T/\left\langle {\dot{q}}^{2}\right\rangle$, and the random force $F_{R}\left(t\right)$ fulfills the relation

[2] $$\begin{array}{c} \left\langle F_{R}\left(t\right)F_{R}\left(0\right)\right\rangle =k_{B}T\Gamma \left(t\right). \end{array}$$

Eq. **2** is exact for the Mori GLE (20), for which U(q) is harmonic, while for nonharmonic U(q), Eq. **2** is approximate (21, 23). The validity of these approximations on m and $F_{R}$ is confirmed later by the accurate agreement of GLE predictions with MD simulations.

A key measure to describe the dynamics of an RC is the mean squared displacement (MSD), defined as $C_{\text{MSD}}\left(t\right)=\left\langle {\left(q\left(0\right)-q\left(t\right)\right)}^{2}\right\rangle$, where ⟨·⟩ denotes the ensemble average. The MSD is often written as $C_{\mathrm{MSD}}\left(t\right)\propto t^{\alpha (t)}$ with a time-dependent exponent α(t). In the free case with U(q)=0 for instantaneous friction Γ(t)∝δ(t), i.e. the Markovian case, the MSD exhibits a ballistic regime defined by α=2 at short times followed by a transition to Brownian diffusion with α=1 at long times. Additional features in the MSD, such as oscillations or subdiffusive scaling (the latter being characterized by α(t)<1), can in principle arise either from non-Markovian friction Γ(t), from features in the free-energy landscape U(q), from coordinate-dependent friction (24–26), or from a combination of these effects. The interplay of these effects and how they lead to subdiffusion in protein conformational dynamics is still debated.

Theories of subdiffusion often postulate power-law memory $\Gamma \left(t\right)\propto t^{-\alpha }$, motivated by the idea that the systems of interest exhibit self-similar structures and hence display fractal patterns in the dynamics on all time scales (15, 27, 28). However, real-world physical systems are naturally limited by a smallest and largest temporal and spatial scale, which necessarily limits the self-similarity (29). This is certainly the case for proteins, which are atomic in composition at their shortest scales and fold into structures that are typically of the order of nanometers in size. Interestingly, proteins are known to exhibit a wide range of relevant time scales, spanning from solvent interactions at the picosecond scale, up to microseconds and even seconds. However, in simulations as well as in experiments this wide range of time scales is often not completely captured, which illustrates why protein folding is often described in terms of fractals and power-law models over all available time scales of the data. Previous theoretical works revealed that power-law memory can be accurately described by sums of very few exponential contributions and that such multiexponential memory contributions produce subdiffusion in the MSD over many orders of magnitude in time (12, 29, 30).

The mean first-passage-time (MFPT) is another key measure that links the conformational dynamics of proteins to their folding and allows us to evaluate folding times. It is defined as the mean time to reach the final RC position $q_{F}$ for the first time from a starting position $q_{S}$. The MFPT from the unfolded to the folded state is called the folding time and vice versa for the unfolding time. Folding and unfolding times of proteins are measurable in experiments, they are strongly influenced by non-Markovian effects (13, 31) and are highly relevant for their biological functioning (32).

In this paper, we extract the friction memory Γ(t) of several fast-folding proteins from published MD simulation trajectories (12, 33). The proteins (λ-repressor, $\alpha_{3}D$, protein-G, and the homopeptide Alanine9), chosen to cover a wide range of free energy barrier heights, all exhibit restricted power-law friction memory for well-established one-dimensional RCs. The friction kernels are all well described by the sum of very few exponential contributions. This multiexponential memory effectively produces power-law behavior for a finite range of time in the MSD. We find that the subdiffusive regime in the MSD is well captured by the GLE with multiexponential memory but cannot be reproduced by Markovian models. Moreover, we show that the GLE describes the MFPTs well for both folding and unfolding. In contrast, Markovian models solely capture the long-time behavior of MSD and MFPT, even if they include coordinate-dependent friction.

The extraction and fit of the friction memory kernels allow us to accurately predict the subdiffusive MSD regime using analytic theory (30), where the memory is multiexponential and individual memory components are hierarchically ordered. In conjunction with GLE simulations, this makes it possible for us to disentangle the influences of the free energy and of the friction on the conformational dynamics of proteins. The observed hierarchical relationship between friction memory components further enables us to estimate memory contributions below the temporal resolution of the data, the inclusion of which leads to an even better description of the MSD. Our results highlight the importance of memory effects in protein conformational and folding dynamics and specifically show that memory dominates the dynamics of fast-folding proteins compared to effects due to the free-energy landscape for a wide time range. Our finding that fast-folding proteins can be described by hierarchically ordered friction-memory components, which leads to subdiffusive conformational dynamics, reconciles the ideas of hierarchical and fractal protein folding theories (34–38).

## 1. Modeling and Results

The folding of proteins without energy consumption is an equilibrium process (39, 40), thus the GLE (Eq. **1**) together with Eq. **2** are appropriate to describe the dynamics of the RC, which is distributed according to the Boltzmann distribution $p\left(q\right)\propto e^{-U\left(q\right)/k_{B}T}$. Inversely, the free energy is extracted from trajectories via

[3] $$\begin{array}{c} U\left(q\right)=-k_{B}T\ln\left(p\left(q\right)\right). \end{array}$$

**Fig. 1.**
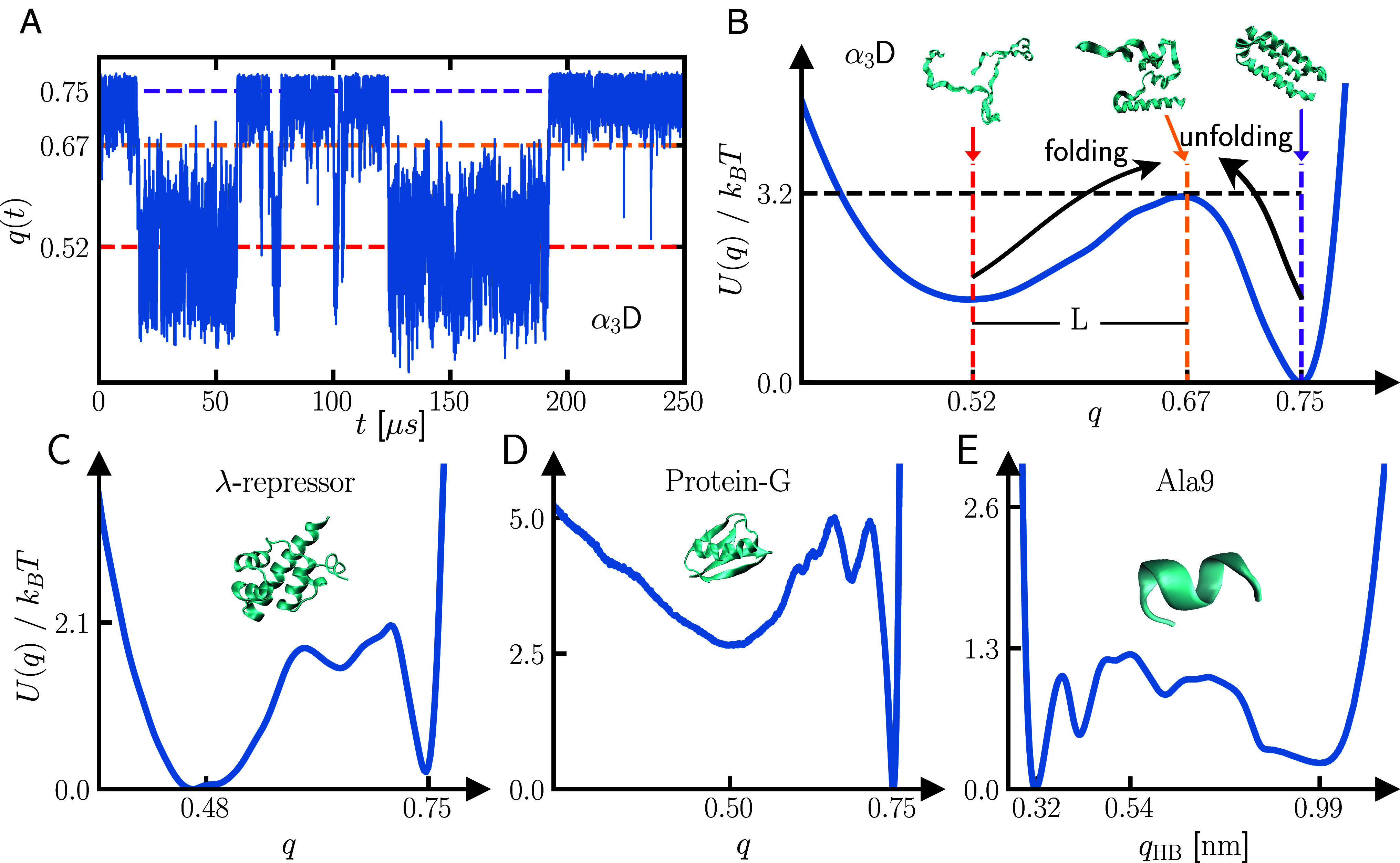
(*A*) Trajectory of the fraction of native contacts RC q(t) from atomistic MD simulations of the $\alpha_{3}$D protein (33) (see *SI Appendix* for details on constructing q(t)). (*B*) The free-energy profile U(q) extracted from the q(t) trajectory for the $\alpha_{3}$D protein, evaluated using Eq. **3**. Colored vertical dashed lines denote the minima and the maximum of U(q), with exemplary snapshots of the folded, unfolded, and barrier-state conformations above the respective RC positions. L denotes the distance between the unfolded state and the barrier state. The extracted free-energy profiles for (*C*) λ-repressor, (*D*) protein-G, and (*E*) Ala9, including snapshots of the folded-state conformations, where $q_{\mathrm{HB}}$ is the ${\mathrm{HB}}_{4}$ RC for Ala9 (12), which has the value $q_{\mathrm{HB}}=0.32\;\mathrm{nm}$ in the folded state.

Fig. 1*A* shows a 250μs trajectory segment for the fraction of native contacts RC taken from an extensive MD simulation of the fast-folding protein $\alpha_{3}D$, which exhibits transitions between the folded and unfolded states every few tens of microseconds (33). Fig. 1*B* depicts the free-energy profile U(q) extracted from such trajectories by Eq. **3**, which exhibits two local minima corresponding to the folded and unfolded state, illustrated by exemplary snapshots of the protein structure at the two minima and the barrier-top. In *SI Appendix*, we demonstrate that barrier states are very diverse.

The free-energy landscapes and native state conformations of all other proteins in this study are shown in Fig. 1*C*–*E*, which exhibit free energy barriers with heights $U_{0}$ between $1\;k_{B}T$ and $5\;k_{B}T$. The proteins are λ-repressor, a transcription inhibitor in bacteriophages (41), protein-G, present in the cell walls of bacteria and involved in antibody binding (42), $\alpha_{3}D$, a designed α-helical fast-folding protein (43), and Ala9, a nine-residue homopeptide that forms a single α-helix (44). The temporal resolution of the MD simulation data of the fast-folding proteins (33) is Δ=0.2ns, the resolution of the Ala9 simulation data is (12) Δ=1fs. It was shown that the temporal resolution of the analyzed MD data leads to valid friction kernels Γ(t), where only the information on times below the discretization is obscured (13, 45). For the fast-folding proteins, we use the fraction of native contacts RC (46), which is a unitless measure of how many residues are within a cutoff distance to their folded-state neighbors (see *SI Appendix* for details). For Ala9, we use the ${\mathrm{HB}}_{4}$ coordinate, which measures the mean length of hydrogen bonds between residues k and k+4 and which is minimal in the α-helical structure (12).

**Fig. 2.**
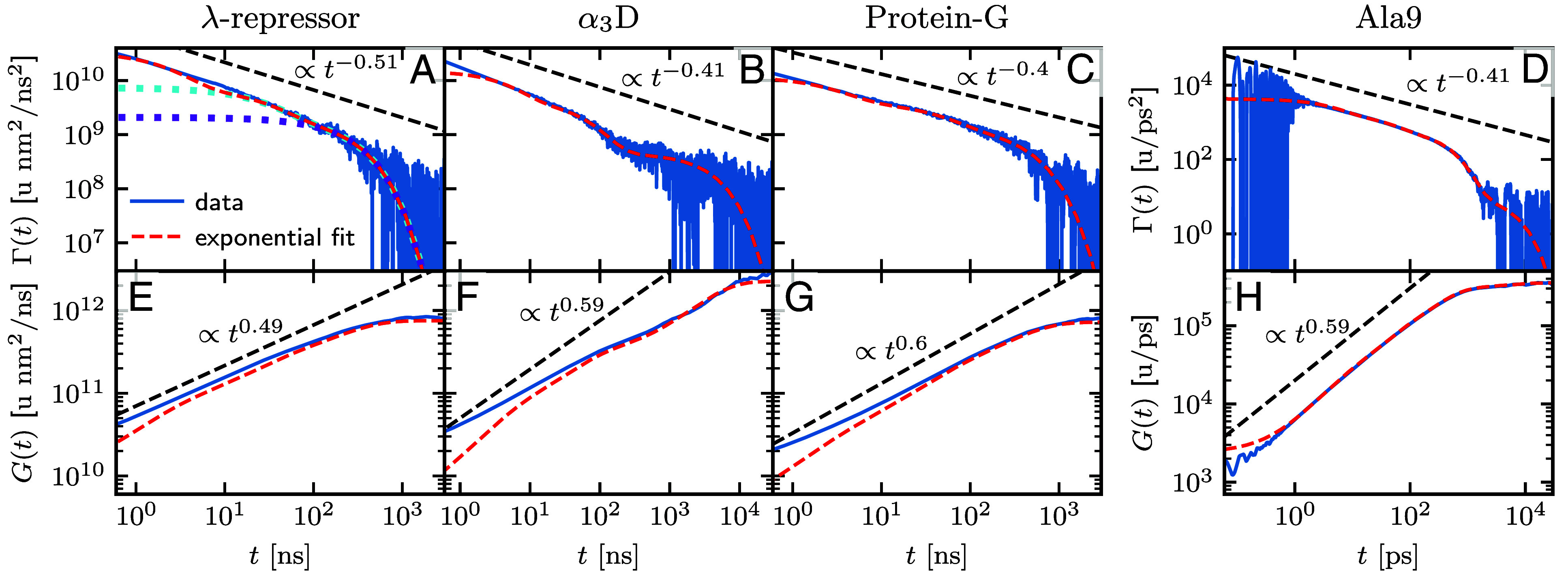
Friction kernels Γ(t) (*A*–*D*) and their corresponding running integrals G(t) (*E*–*H*), extracted from MD simulations for λ-repressor, $\alpha_{3}$D, protein-G (33) and Ala9 (12), respectively (blue lines). The straight black dashed lines represent power-law behavior with values of $\alpha_{\mathrm{sub}}^{\mathrm{data}}$, extracted from the MSD as an average over Eq. **8** shown in Table 1. The red dashed lines represent the multiexponential fit to the friction kernel Γ(t) in Eq. **4** using n=5 exponentials for Ala9 and n=3 exponentials for all other proteins. The dotted lines in (*A*) indicate contributions from the individual exponential components in Γ(t) (Eq. **4**). The dashed red lines in (*E*–*H*) result from integration of the corresponding red dashed lines in (*A*–*D*), according to Eq. **5**.

We extract the friction kernel Γ(t) in the GLE Eq. **1** from trajectories using previously established methods (12, 13, 47, 48) (see *SI Appendix* for details). In Fig. 2*A*–*D*, we show that, for all proteins, the friction kernels show scaling behavior $\Gamma \left(t\right)\propto t^{-\alpha_{\mathrm{sub}}}$ with $0.40\leq \alpha_{\mathrm{sub}}\leq 0.51$ on intermediate times and exhibit exponential decay for long times. In fact, the friction kernels are well described by a multiexponential fit of the form

[4] $$\begin{array}{c} \Gamma \left(t\right)=\sum_{i=1}^{n}\frac{\gamma_{i}}{\tau_{i}}e^{-t/\tau_{i}}\;, \end{array}$$

with n=3 components for the fast-folding proteins and n=5 components for the homopeptide Ala9. All friction coefficients $\gamma_{i}$ and memory times $\tau_{i}$ are given in *SI Appendix*. Importantly, we observe clear power-law behavior in Γ(t) over two decades in time, even with the inclusion of only three exponential terms. We show in *SI Appendix* that the extracted friction kernel from several well-established RCs are different but exhibit similar power-law behavior for λ-repressor.

The finite length of this power-law behavior becomes especially apparent for the integrated friction G(t), which is defined as

[5] $$\begin{array}{c} G\left(t\right)=\int_{0}^{t}\Gamma \left(s\right)ds. \end{array}$$

For a pure power-law friction kernel $\Gamma \left(t\right)\propto t^{-\alpha_{\mathrm{sub}}}$ one obtains $G\left(t\right)\propto t^{1-\alpha_{\mathrm{sub}}}$. In Fig. 2*E*–*H* it is seen for all proteins, that G(t) (blue lines) exhibits power-law behavior only over a finite time range. For long times, G(t) is well described by the integral over the multiexponential Eq. **4** (red broken lines). In the long time limit, the integrated kernel reaches a plateau, which corresponds to the total friction

[6] $$\begin{array}{c} \gamma_{\mathrm{tot}}=G\left(\infty \right)=\sum_{i=1}^{n}\gamma_{i}. \end{array}$$

We use $\gamma_{\mathrm{tot}}$ to define the inertial time scale $\tau_{m}=m/\gamma_{\mathrm{tot}}$, which, for proteins, is much smaller than the diffusion time $\tau_{D}=\gamma_{\mathrm{tot}}L^{2}/k_{B}T$. Here, $\tau_{D}$ is the time that the RC needs to diffusive over the distance L for a flat free-energy profile, where L is the distance in the RC space between the unfolded state and the barrier-top, as depicted in Fig. 1*B*. In Table 1, we present the ratios of $\tau_{m}/\tau_{D}$ for the different proteins. These values are very small, indicating that the RC dynamics are strongly overdamped for all proteins.

**Table 1.** Free energy barrier height $U_{0}$ relative to the global minimum, ratio of inertial time and diffusion time $\tau_{m}/\tau_{D}$, mean values for memory time ratio c and friction coefficient ratio d, as defined in Eq. **7**, and friction coefficients $\gamma_{i+1}/\gamma_{i}$ defined in Eq. **4**

Protein	$U_{0}/k_{B}T$	$\tau_{m}/\tau_{D}$	c	d	$\alpha_{\mathrm{sub}}^{\mathrm{pred}}$	$\alpha_{\mathrm{sub}}^{\mathrm{data}}$	$\gamma_{3}/\gamma_{2}$	$\gamma_{2}/\gamma_{1}$
λ-repressor	2.1	$2.6\times 10^{-7}$	12	2.7	0.59±0.04	0.51±0.02	3.7	2.5
$\alpha_{3}$D	3.2	$3.9\times 10^{-8}$	21	5.3	0.44±0.02	0.41±0.06	8.2	4.4
Protein-G	5.0	$2.1\times 10^{-7}$	14	5.1	0.38±0.03	0.40±0.04	5.1	5.2
Ala9	1.3	$1.4\times 10^{-9}$	9	4.0	0.38±0.04	0.41±0.06	3.5	5.5

The subdiffusive scaling prediction $\alpha_{\mathrm{sub}}^{\mathrm{pred}}$ from Eq. **9** is compared to the extracted scaling exponent of the MD data $\alpha_{\mathrm{sub}}^{\mathrm{data}}$. The latter is obtained as a logarithmic time average of α(t) defined in Eq. **8**, i.e., an average using exponentially spaced time points, in the regime $\tau_{1}<t<\tau_{2}$ for λ-repressor, $\alpha_{3}$D, and protein-G, and in the regime $\tau_{2}<t<\tau_{4}$ for Ala9. The complete sets of extracted memory kernel parameters are given in *SI Appendix*.

For friction coefficients $\gamma_{i}$ and memory times $\tau_{i}$ that are hierarchically ordered and exponentially spaced with ratios c and d according to

[7] $$\begin{array}{c} \begin{array}{cc} \tau_{i} & =\tau_{1}c^{i-1}\\ \gamma_{i} & =\gamma_{1}d^{i-1} \end{array} \end{array}$$

the friction kernel in Eq. **4** appears to satisfy a power law $\Gamma \left(t\right)\propto t^{-\alpha_{\mathrm{sub}}}$ over a finite range of time, where the range increases with an increasing number of exponential components (29, 30). Interestingly, we find that the structure of the fitted memory components matches rather closely the hierarchical structure defined in Eq. **7**, as indicated by the rather equal ratios of friction coefficients for each protein in Table 1 (the complete set of parameters is given in *SI Appendix*). This leads to an even spacing between individual memory components on the log–log scale shown by the cyan, violet, and red lines in Fig. 2*A*. The exponent in the friction kernel $\Gamma \left(t\right)\propto t^{-\alpha_{\mathrm{sub}}}$ translates directly into a subdiffusive scaling regime in the MSD, with $C_{\mathrm{MSD}}\left(t\right)\propto t^{\alpha_{\mathrm{sub}}}$ for intermediate times (29, 30, 49). The time-dependent scaling exponent is defined as the logarithmic derivative of the MSD as

[8] $$\begin{array}{c} \alpha \left(t\right)=\frac{d\;\ln(C_{\mathrm{MSD}}\left(t\right))}{d\;\ln(t)}, \end{array}$$

where the intermediate subdiffusive scaling for multiexponential memory with c>d and d>1 for times $\tau_{1}<t<\tau_{n}$ is predicted as (30)

[9] $$\begin{array}{c} \alpha_{\mathrm{sub}}^{\mathrm{pred}}=\frac{\ln(c/d)}{\ln(c)}=\frac{\ln\left({\left(\frac{\gamma_{1}\tau_{i}}{\tau_{1}\gamma_{i}}\right)}^{\frac{1}{i-1}}\right)}{\ln\left({\left(\frac{\tau_{i}}{\tau_{1}}\right)}^{\frac{1}{i-1}}\right)}. \end{array}$$

**Fig. 3.**
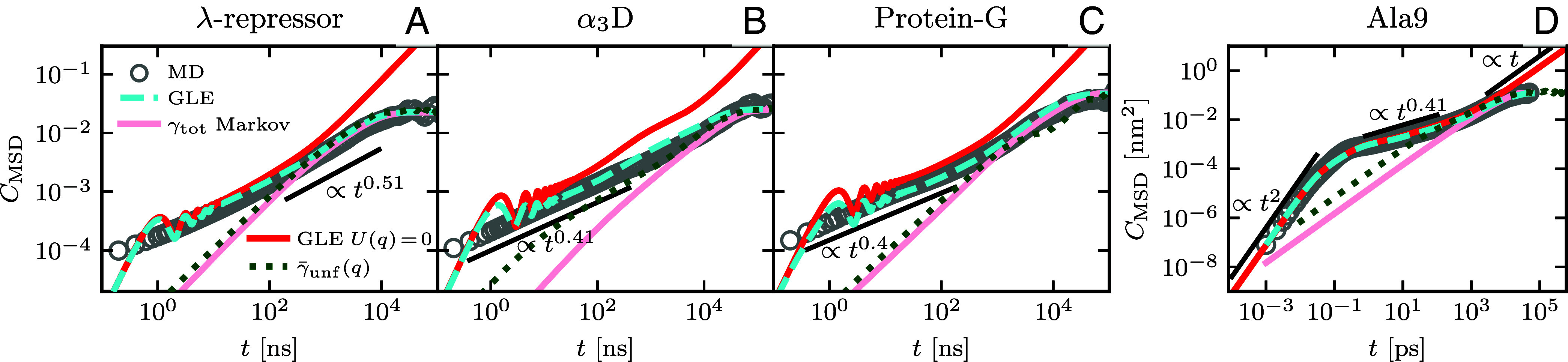
(*A*–*D*) MSDs for different proteins. Data extracted from MD simulations (12, 33) are shown as gray circles. The cyan dashed lines represent the GLE simulation result using the fitted kernel Γ(t) of Fig. 2*A*–*D* and the free-energy landscape U(q) in Fig. 1*B*–*E*. The red lines are the analytical GLE prediction for U(q)=0 (30). The dotted lines result from simulations of Eq. **10** with Markovian coordinate-dependent friction profiles ${\overset{¯}{\gamma }}_{\mathrm{unf}}\left(q\right)$ extracted from the unfolding MFPT profile of the MD data and shown in *SI Appendix*. The pink lines originate from simulations of the Markovian limit of the GLE using the constant total friction $\gamma_{\mathrm{tot}}$ and U(q). Simulation details are given in *SI Appendix*.

In Table 1, we compare the subdiffusive scaling exponent $\alpha_{\mathrm{sub}}^{\mathrm{pred}}$ to the observed subdiffusive scaling in the MSD $\alpha_{\mathrm{sub}}^{\mathrm{data}}$. The former is predicted by Eq. **9** using friction components $\gamma_{i}$ and memory times $\tau_{i}$ from the fit of the memory kernels in Fig. 2, for which $\tau_{i}$ are within the subdiffusive regime, as described in detail in *SI Appendix*; $\alpha_{\text{sub}}^{\text{data}}$, is computed as a logarithmic time average of α(t) in the subdiffusive regime using Eq. **8**. The observed scaling exponents $\alpha_{\mathrm{sub}}^{\mathrm{data}}$ agree well with the scaling exponent $\alpha_{\mathrm{sub}}^{\mathrm{pred}}$, where the prediction of the subdiffusive scaling via Eq. **9** does not include effects due to the free-energy landscape (30). This suggests that the memory alone dictates the subdiffusive scaling in the MSD of fast-folding protein dynamics. In Fig. 3, we show the MSD of all proteins from MD simulations as gray circles. The MSD from simulations of the GLE Eq. **1** (cyan broken line) describes the MSD from MD data very well for all proteins on a double-logarithmic scale. Here, it should be noted that the oscillations on short times in the MSD from GLE simulations are due to unresolved memory effects at times below the temporal resolution of the available data, as we show later. For simulations of the GLE, we use the extracted free-energy landscape U(q) in Fig. 1*B*–*E*, the fitted multiexponential friction kernels in Fig. 2*A*–*D* and a Markovian embedding scheme (12, 29, 50, 51). For Ala9 in Fig. 3*D*, the temporal resolution is higher (Δ=1fs compared to Δ=0.2ns for the other proteins), which leads to a perfect agreement between the GLE simulation and the MD data for all times, even resolving the short-time ballistic regime, where α=2.

Markovian Langevin simulations using $\Gamma \left(t\right)=2\gamma_{\mathrm{tot}}\delta \left(t\right)$ and the free-energy profiles U(q) extracted from the MD simulations (pink lines in Fig. 3), do not reproduce the subdiffusive scaling behavior at intermediate times. Even when generalizing the overdamped Langevin equation to include coordinate-dependent friction γ(q) according to ref. 25

[10] $$\begin{array}{c} 0=-\nabla U\left(q\right)-\gamma \left(q\right)\dot{q}-\frac{k_{B}T}{2}\frac{\gamma^{\prime }\left(q\right)}{\gamma (q)}+\sqrt{k_{B}T\gamma \left(q\right)}\xi \left(t\right), \end{array}$$

the resulting MSDs (dotted lines in Fig. 3) deviate strongly from the MD MSDs. In *SI Appendix*, we show that the inclusion of inertial effects in Eq. **10** does not improve the agreement with the MD results.

**Fig. 4.**
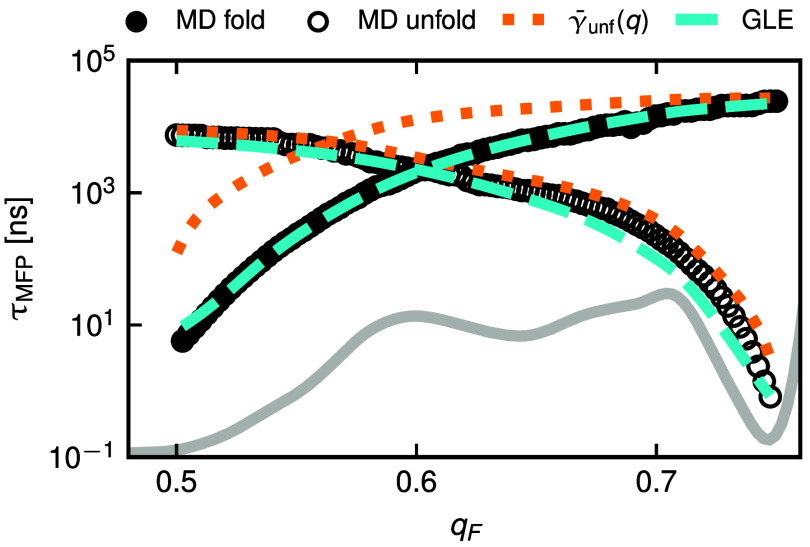
MFPT profiles from MD simulations of λ-repressor in the folding direction, starting from $q_{S}=0.5$ (filled black circles), and in the unfolding direction starting from $q_{S}=0.75$ (open black circles). The gray line shows the free-energy profile U(q). Predictions are shown in cyan from simulations of the GLE Eq. **1** and in orange from the analytic solution of the Langevin Eq. **10**, including the coordinate-dependent friction profile ${\overset{¯}{\gamma }}_{\mathrm{unf}}\left(q\right)$ extracted from the MD MFPT in the unfolding direction by averaging over different starting positions. We calculate MFPTs from first-first-passage events, see *SI Appendix* for details on MFPTs and γ(q) extraction.

The GLE captures not only the MD MSD but also the MD MFPT with high accuracy, as exemplified in Fig. 4 for λ-repressor, where we show folding and unfolding MFPT profiles. In contrast, the folding dynamics are not consistently captured by a Markovian model: The MFPT prediction of Eq. **10** (dotted orange line) agrees well with the unfolding MD data (open black circles), since the friction profile is constructed from the unfolding MD MFPT data. However, the prediction of Eq. **10** deviates significantly from the MFPT of the MD data in the folding direction (filled black circles) for all but the longest times. This is expected since the diffusion times of the proteins are much longer than the memory decay time (see *SI Appendix*), in which case non-Markovian barrier-crossing speed-up is insignificant (20). In turn, this means that Markovian models can be used to extract friction properties of non-Markovian systems on time scales that exceed the longest memory time (25, 26). Additional details on all MFPT profiles and coordinate-dependent friction profiles γ(q) are given in *SI Appendix*.

**Fig. 5.**
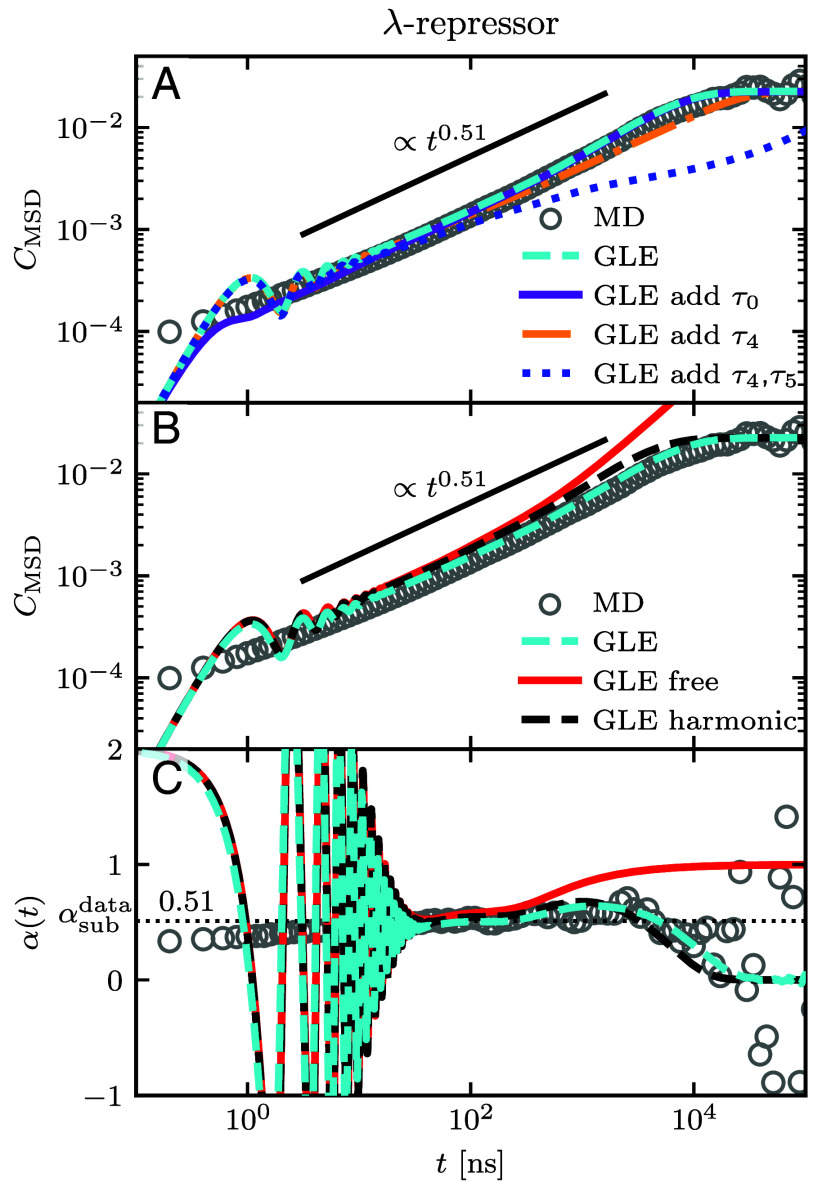
λ-repressor MSD and scaling exponent modeled by the GLE. (*A*) MSD from MD simulations (33) (gray circles) compared to various GLE simulations. The dashed cyan line represents the result with the extracted U(q) and fitted Γ(t). The violet, orange, and blue lines result from GLE simulations with additional memory components on shorter time scale $\tau_{0}$, longer time scale $\tau_{4}$ and two longer time scales $\tau_{4}$, $\tau_{5}$, respectively, according to Eq. **4** with values of c and d in Eq. **7** taken from Table 1. (*B*) The black dashed line is the analytical result for the MSD in a harmonic potential $U\left(q\right)=Kq^{2}/2$ with $K=k_{B}T/\left\langle {\left(q-\left\langle q\right\rangle \right)}^{2}\right\rangle$. (*C*) Time-dependent exponent extracted by Eq. **8** from the lines shown in (*B*). The horizontal dotted line shows the average of the MD data over intermediate times, which is $\alpha_{\mathrm{sub}}^{\mathrm{data}}=0.51$ given in Table 1.

For short times, the MSDs from GLE simulations, parameterized by the fitted kernels, exhibit nanosecond-scale oscillations (Fig. 3*A*–*C*), which are absent in the MD MSD. These oscillations are reduced by adding an exponential component to the memory kernel (Eq. **4**) at shorter times, where $\tau_{i}$ and $\gamma_{i}$ of the additional memory term are extrapolated by the average ratios c and d, defined in Eq. **7** and given in Table 1. This reduction of oscillations is seen by comparing the violet and cyan lines in Fig. 5*A* and it further improves the agreement with the MSD of the MD data. The addition of memory components on shorter time scales leaves the total friction $\gamma_{\mathrm{tot}}$ virtually unchanged, since c>1 and d>1 lead to exponentially smaller friction coefficients for the short memory times. The improvement of the MSD description while conserving the total friction suggests that there are likely more exponential components present on time scales below the temporal resolution of the data (see *SI Appendix* for results for all other proteins). This further indicates that the hierarchy of the memory components, Eq. **7**, is appropriate to describe protein conformational dynamics. On the contrary, adding memory components according to the observed hierarchy on longer time scales increases $\gamma_{\mathrm{tot}}$ and leads to deviations compared to the MSD of the MD data. While adding one memory component on a longer time scale (orange line in Fig. 5*A*) has only a minor effect, the addition of two memory components on longer time scales (dark-blue dotted line) clearly leads to strong deviations from the MD data (more detail in *SI Appendix*). This implies that the longest time scale of the memory is resolved by our fit of Γ(t), whereas shorter memory times are involved in the fast-folding protein dynamics that are not resolved due to the finite time resolution of the available data. Previously developed optimization methods (45) represent an alternative method to determine time scales below the temporal resolution of the data that is independent of the observed ratios c and d.

Deviations between the analytically predicted MSD from the GLE for U(q)=0 and the MSD of the MD data increase for increasing barrier height $U_{0}$ (Table 1), as shown in Fig. 3. For λ-repressor and Ala9, with maximal barrier heights of $2.1\;k_{B}T$ and $1.3\;k_{B}T$, respectively, the dynamics for short and intermediate times are dictated by memory alone and the main influence of the free-energy landscape is the long-time confinement. Therefore, the analytical prediction of the MSD with multiexponential memory Eq. **4** in a harmonic potential, adjusted to produce the same positional variance as the free energy U(q), captures the dynamics of λ-repressor and Ala9 well for all times, as shown for λ-repressor by the black dashed line in Fig. 5*B*. Moreover, the time-dependent scaling behavior α(t) of the MD data extracted via Eq. **8** (gray circles) is well captured by the description of the GLE with a harmonic potential (dashed black line), as shown in Fig. 5*C* for λ-repressor and in *SI Appendix* for all proteins. For higher free-energy barriers, as exhibited by $\alpha_{3}$D ($3.2\;k_{B}T$) and protein-G ($5.0\;k_{B}T$), the MSD for the analytical prediction with U(q)=0 appears shifted upward compared to the MD result, i.e. the red lines are higher than the gray circles in Fig. 3 *B* and *C* at intermediate times. Thus, the accurate prediction of the MSD of $\alpha_{3}$D and protein-G requires both, the knowledge of the free-energy profile and the memory. Nevertheless, the intermediate subdiffusive scaling is still captured well by $\alpha_{\text{sub}}^{\text{pred}}$ in Eq. **9** (shown in Table 1), which includes no information about U(q). This further implies that memory governs the subdiffusion of protein conformational dynamics.

## 2. Discussion and Conclusions

We extract the friction memory kernel Γ(t) of different fast-folding proteins from MD simulations and find that Γ(t) is well described as a multiexponential, as shown in Fig. 2. In previous work, it has been suggested that protein folding is fractal and thus exhibits power-law behavior and subdiffusion across all scales (14, 15, 36, 38). However, it has also been argued that proteins fold hierarchically (34, 35). We show that fast-folding protein conformational dynamics are well described by the sum of a small number (n=3 to 5) of exponential memory components, which leads to effective power-law behavior in the memory kernel and the MSD over a finite time range. In fact, the memory components follow a hierarchical pattern with approximately constant ratios of friction coefficients and memory times (Table 1 and *SI Appendix*) Eq. **7**, which gives rise to a pronounced subdiffusive regime in the MSD. The hierarchical structure of the memory in the fraction of native contacts RC is possibly related to hierarchical folding mechanisms. This would mean that individual memory components are related to the dynamics of differently sized subunits of the protein which move on different time scales. In order to test this, one could use amino acid mutation studies in conjunction with coarse-grained or MD simulations to connect the memory contributions to structural and environmental features. For instance, it was recently shown that by reducing pH and thereby eliminating salt-bridge interactions between tertiary structures, contributions from the long timescale friction components are dramatically reduced (19).

Importantly, the extraction of friction kernels and application of the GLE framework only requires an RC trajectory and not the full atomistic information available from MD simulations. Thus, it can be readily applied to experimental time-series data from force spectroscopy (52) or FRET spectroscopy (53–55). These techniques also enable the extraction of single-molecule dynamical information like transition-path times, which were shown to strongly depend on friction memory in simulations, especially in the overdamped limit relevant for protein folding dynamics (56). Here, the GLE framework could significantly improve the description of experiments compared to Markovian models. GLE simulations are computationally much cheaper than MD simulations and as we show in this work, the GLE accurately describes protein-folding dynamics in terms of the MSD (Fig. 3) and MFPT (Fig. 4 and *SI Appendix*). Putatively, friction kernel parameters extracted from experiments can be used in efficient GLE simulations to predict dynamical features on time scales that are not accessible in MD simulations or experiments. Furthermore, the description of subdiffusive behavior by multiexponential friction memory in the GLE is transferable to other systems, for instance to cell motion (57–59), polymer network dynamics (60, 61), and even weather data (62).

Memory effects dominate the subdiffusive behavior of fast-folding proteins, more so than contributions from the free-energy landscape. This is seen by comparing GLE simulations with and without memory using the free-energy landscape U(q) (Fig. 3), where only the long-time MSD behavior that reflects confinement due to U(q) is captured by Markovian models. For proteins with small free energy barriers up to ∼2 $k_{B}T$, the MSD is well captured by the description with the GLE and U(q)=0 up to the longest memory time. In fact, the subdiffusive MSD scaling α(t) is well captured by the GLE in a harmonic potential for all times (Fig. 5 *B* and *C* and *SI Appendix*). Further, the scaling exponent $\alpha_{\mathrm{sub}}^{\mathrm{data}}$ in the subdiffusive MSD regime is accurately predicted by Eq. **9**, which is independent of the free-energy landscape. This underscores the importance of memory effects for the dynamics compared to effects due to barriers or the specific shape of the free-energy landscape. We expect that for proteins with significantly higher free energy barriers than those considered in this study, memory effects are still crucial to describe the dynamics, but note that additional subdiffusive regimes in the MSD can arise from barrier-crossing processes (30, 63, 64).

Neither the Markovian description with constant friction $\gamma_{\text{tot}}$ nor the assumption of coordinate-dependent Markovian friction γ(q) Eq. **10** reproduce subdiffusive scaling in the MSD on intermediate time scales (Figs. 3 and 5). The coordinate-dependent friction γ(q) can also not consistently describe MFPT profiles (Fig. 4 and *SI Appendix*). Overall, the GLE accurately describes the RC dynamics of protein folding, whereas Markovian models fail to fully capture the dynamics.

## 3. Methods

The native state of a protein and the fraction of native contacts RC are defined in *SI Appendix*, section I. An iterative formula for extracting the friction kernel from discrete MD simulation trajectories is derived in *SI Appendix*, section III. The relationship between coordinate-dependent friction and the MFPT is discussed in *SI Appendix*, section VI, the calculation of averaged friction profiles is explained in *SI Appendix*, section VII. Finally, *SI Appendix*, section VIII outlines the framework for simulating the Langevin equation with coordinate-dependent friction and inertial effects.

## Supplementary Material

Appendix 01 (PDF)

## Data Availability

The code to simulate the GLE and position dependent LE are available at https://github.com/kanton42/msd_subdiffusion (50) and kernel extraction methods at https://github.com/lucastepper/memtools (48).
