# Supplementary material for: Hierarchical friction memory leads to subdiffusive configurational dynamics of fast-folding proteins
Source: Proc Natl Acad Sci U S A. 2026 Feb 6;123(6):e2516506123. doi: 10.1073/pnas.2516506123 (PMC12890985; doi:10.1073/pnas.2516506123)
Supplement: Supplementary file 1 — Appendix 01 (PDF) [file pnas.2516506123.sapp.pdf]

Supporting information: Hierarchical friction memory leads to subdiffusive configurational dynamics of fast-folding proteins

Anton Klimek, Benjamin A. Dalton, Lucas Tepper, Roland R. Netz

I Fraction of native contacts reaction coordinate

To calculate the fraction of native contacts reaction coordinate q, we follow Best *et al.* [1] (see also [2,3]). For each protein, we project the backbone C_α positions from the all-atom trajectories onto the one-dimensional q coordinate. To define the native state, we first compute the root-mean-squared deviation (RMSD) between each pair of configurations that occur in the simulation, using Eq. (S15). We then count the number of configurations that lie within 0.2 nm of RMSD for every configuration and the native state is defined as the configuration with the highest count. In the assigned native state, all Cα pairs separated by at least 5 residues in sequence and less than 0.9 nm in Cartesian distance are defined as native contacts. Each protein has $N_{\rm nc}$ such native contacts with separation vectors in the native state $\mathbf{s}_{ij}^0$, which have magnitudes $s_{ij}^0 = \sqrt{\mathbf{s}_{ij}^0 \cdot \mathbf{s}_{ij}^0}$. At time t, the separation vectors are denoted as $\mathbf{s}_{ij}(t)$, with magnitudes $s_{ij}(t) = \sqrt{\mathbf{s}_{ij}(t) \cdot \mathbf{s}_{ij}(t)}$. The fraction of native contacts present at time t is

$$q(t) = \frac{1}{N_{\rm nc}} \sum_{i<j} \frac{1}{1 + \mathrm{e}^{\beta(s_{ij}(t) - \gamma s_{ij}^0)}}, \tag{S1}$$

where the sum runs over native contact pairs, with parameters $\beta = 3\,\mathrm{nm}^{-1}$ and $\gamma = 1.6$.

II Examples of configurations at the barrier for α_3D

In Fig. 1B of the main manuscript, we show example simulation snapshots of the α3D protein in the folded, unfolded, and barrier-top states. In the shown barrier configuration, one α-helix has already formed, while the remainder of the protein is disordered. In Fig. S1 we show eight randomly chosen barrier state configurations for α_3D. We observe a wide range of barrier configurations, all corresponding to the same q value.

III Extraction of the friction memory kernel

To determine the memory kernel $\Gamma(t)$ from the GLE given by Eq. 1 in the main text, we use the running integral extraction scheme for general potentials [4]. The running integral $G(t)$ is given by

$$G(t) = \int_0^t dt' \Gamma(t')\,. \tag{S2}$$

Correlation functions are constructed by taking the ensemble average of the GLE (Eq. 1 in main text), multiplied by the initial position of the reaction coordinate $q(0)$,

$$m\Big\langle q(0)\ddot{q}(t)\Big\rangle = -\int_0^t \Big\langle \Gamma(t-t') q(0) \dot{q}(t') dt' \Big\rangle - \Big\langle q(0) \nabla U\big[q(t)\big]\Big\rangle + \Big\langle q(0) F_R(t)\Big\rangle, \tag{S3}$$

and with the initial velocity of the reaction coordinate $\dot{q}(0)$,

$$m\Big\langle \dot{q}(0)\ddot{q}(t)\Big\rangle = -\int_0^t \Big\langle \Gamma(t-t') \dot{q}(0) \dot{q}(t') dt' \Big\rangle - \Big\langle \dot{q}(0) \nabla U\big[q(t)\big]\Big\rangle + \Big\langle \dot{q}(0) F_R(t)\Big\rangle. \tag{S4}$$

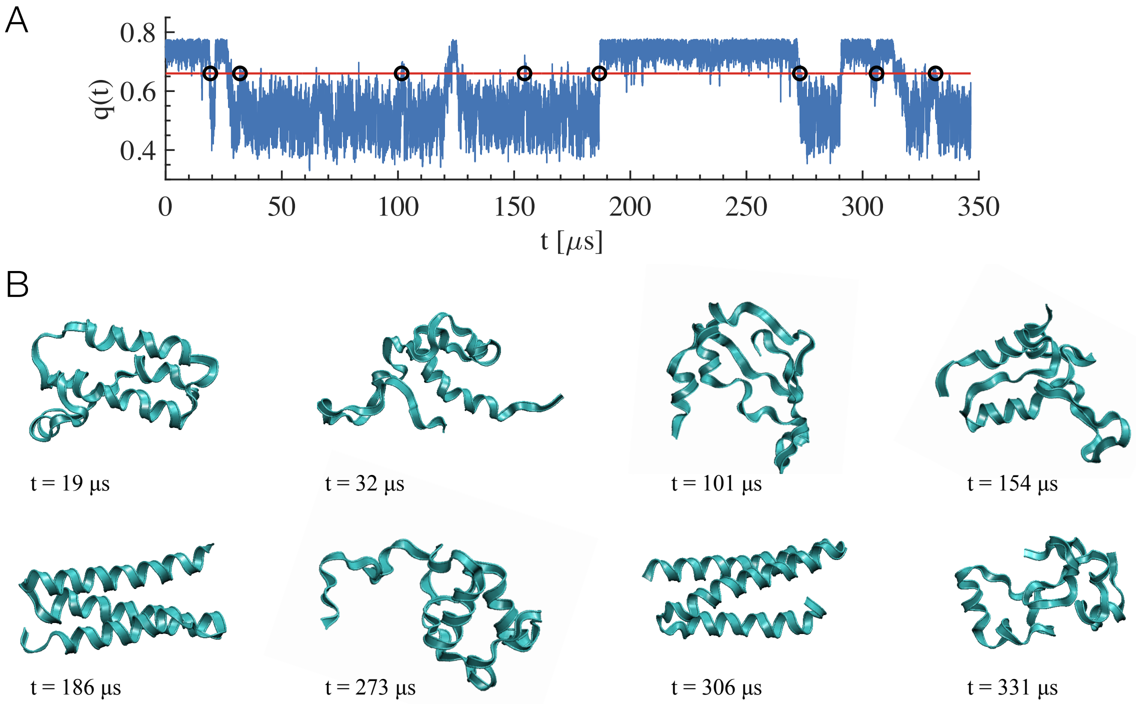

Figure S1: (A) Trajectory segment for α_3D with eight barrier configurations ($q \sim 0.67$) indicated. These configurations come from folding and unfolding transition paths, as well as from failed attempts to cross the barrier. (B) Eight representative snapshots of the α_3D protein at barrier configurations, corresponding to the trajectory points highlighted in (A).

Since both $\big\langle q(0)F_R(t)\big\rangle = 0$ and $\big\langle \dot{q}(0)F_R(t)\big\rangle = 0$, we can write Eqs. S3 and S4 in terms of the following correlation functions: $C^{q\dot{q}}(t) = \big\langle q(0)\dot{q}(t)\big\rangle$, $C^{\dot{q}\dot{q}}(t) = \big\langle \dot{q}(0)\dot{q}(t)\big\rangle$, $C^{q\nabla U}(t) = \big\langle q(0)\nabla U\big[q(t)\big]\big\rangle$, and $C^{\dot{q}\nabla U}(t) = \big\langle \dot{q}(0)\nabla U\big[q(t)\big]\big\rangle$:

$$m\frac{d}{dt}C^{q\dot{q}}(t) = -\int_0^t \Gamma(t')C^{q\dot{q}}(t-t')dt' - C^{q\nabla U}(t), \tag{S5}$$

$$m\frac{d}{dt}C^{\dot{q}\dot{q}}(t) = -\int_0^t \Gamma(t')C^{\dot{q}\dot{q}}(t-t')dt' - C^{\dot{q}\nabla U}(t). \tag{S6}$$

Integrating Eq. S6 in the time domain, we obtain an equation in terms of $G(t)$,

$$mC^{\dot{q}\dot{q}}(t) - mC^{\dot{q}\dot{q}}(0) = -\int_0^t G(t-t')C^{\dot{q}\dot{q}}(t'')dt'' + C^{q\nabla U}(t) - C^{q\nabla U}(0). \tag{S7}$$

Using that $\frac{d}{dt}C^{q\dot{q}}(t) = C^{q\ddot{q}}(t) = -C^{\dot{q}\dot{q}}(t)$, we evaluate Eq. S5 at $t = 0$, and obtain $mC^{\dot{q}\dot{q}}(0) = C^{q\nabla U}(0)$. It follows that

$$\frac{C^{\dot{q}\dot{q}}(t)}{C^{\dot{q}\dot{q}}(0)}C^{q\nabla U}(0) = C^{q\nabla U}(t) - \int_0^t G(t-t')C^{\dot{q}\dot{q}}(t'')dt''. \tag{S8}$$

By discretizing Eq. S8 using the trapezoidal rule, we arrive at the numerical scheme to extract $G(t)$ directly from the trajectory of a given RC, which reads

$$G_i = \begin{cases} 0, & i = 0 \\ \frac{2}{\Delta C_0^{\dot{q}\dot{q}}}\left[C_1^{\nabla U q} - \frac{C_0^{\nabla U q}}{C_0^{\dot{q}\dot{q}}}C_1^{\dot{q}\dot{q}}\right], & i = 1 \\ \frac{2}{\Delta C_0^{\dot{q}\dot{q}}}\left[C_i^{\nabla U q} - \frac{C_0^{\nabla U q}}{C_0^{\dot{q}\dot{q}}}C_i^{\dot{q}\dot{q}} - \Delta\sum_{j=1}^{i-1} G_j C_{i-j}^{\dot{q}\dot{q}}\right], & i > 1, \end{cases} \tag{S9}$$

where $C_i^{\dot{q}\dot{q}}$ and $C_i^{\nabla U q}$ are calculated from the time series data. $\Gamma(t)$ is then calculated using mid-point numerical differentiation such that $\Gamma_i = (G_{i+1} - G_{i-1})/2\Delta$, with the first and last values given by $\Gamma_1 = (G_2 - G_1)/\Delta$ and $\Gamma_N = (G_N - G_{N-1})/\Delta$, respectively, for a kernel with N values.

IV Fitting-parameter results & simulation details

In this section, we tabulate all parameters of the multi-exponential memory Eq. (4) fitted to the extracted friction kernel $\Gamma(t)$ via least-squares fits. Additionally, we explain the simulation setup of the GLE simulations and the Markovian Langevin simulations with coordinate-dependent friction.

In Tab. S1, we show all characteristic times of the configurational dynamics of the studied proteins. The longest memory time and the first moment of memory times, $\tau_{\text{mem}} = \int_0^\infty t\Gamma(t)dt / \int_0^\infty \Gamma(t)dt$, are on the order of 10 to 100 times smaller than the folding and unfolding MFPT, $\tau_{\text{MFP}}^{\text{fol}}$ and $\tau_{\text{MFP}}^{\text{unf}}$. Nevertheless, the memory strongly influence the dynamics of the MSD, shown in the main text, Fig. 3, and of the MFPT, shown in Fig. 4 and Fig. S3. In Tabs. S3 and S4, we show the friction amplitudes, γ_i, from the multi-exponential fits to the friction kernels in the main text, Fig. 2

The simulation time steps h are, for Ala9, $h = 100\tau_m$ for coordinate-dependent friction Langevin simulations and $h = 10\tau_m$ for GLE simulations and the Langevin simulation with constant friction γ_{tot}. All simulations for the other proteins are performed with $h = \tau_m$. The initial position and velocity are drawn from the respective Boltzmann distribution, and 1000 independent trajectories of length $10^8 h$ are generated to extract average MSD and average MFPT profiles. The MSD of each individual trajectory is extracted via time averaging according to

$$C_{\text{MSD}}(ih) = \frac{1}{N-i+1} \sum_{j=0}^{N-i} (x_{j+i} - x_j)^2 \,, \tag{S10}$$

For the integration of all equations of motion, we use a fourth-order Runge-Kutta integrator, where coordinate-dependent quantities, such as the free energy $U(q)$ or the friction profile $\gamma(q)$ in the case of coordinate-dependent friction, are interpolated by cubic splines in order to obtain values at positions between bins.

The values of c and d given in the main text, Tab. 1, result from the inversion of Eq. (7) using the values of γ_i and τ_i from the fits as

$$c = \left(\frac{\tau_i}{\tau_2}\right)^{\frac{1}{i-2}}$$
$$d = \left(\frac{\gamma_i}{\gamma_2}\right)^{\frac{1}{i-2}} \,, \tag{S11}$$

for Ala9, where we average over $i = 3, 4$ and

$$c = \left(\frac{\tau_i}{\tau_1}\right)^{\frac{1}{i-1}}$$
$$d = \left(\frac{\gamma_i}{\gamma_1}\right)^{\frac{1}{i-1}} \,, \tag{S12}$$

where we average over $i = 2, 3$ for all other proteins, since these include the memory times τ_i that lay within the subdiffusive regime of the MSD. Here, the values γ_i and τ_i are the fit parameters given in Tabs. S1, S3, S4. Consequently, values of $\alpha_{\text{sub}}^{\text{pred}}$ are obtained by averaging Eq. (9) using the c and d values given in Eq. (S11) for Ala9 with $i = 3, 4$ and using Eq. (S12) for all other proteins with $i = 2, 3$; errors are estimated via the standard deviation. The ratios of consecutive memory times and consecutive friction coefficients are shown in Tab. S2 to exhibit similar values across all scales and even across a few different proteins. These constant ratios lead to an even spacing between individual memory components on the log-log scale of t and $\Gamma(t)$. This is depicted in Fig. 2A of the main text, where the violet line corresponds to $\frac{\gamma_3}{\tau_3} e^{-t/\tau_3}$, the cyan line corresponds to $\frac{\gamma_3}{\tau_3} e^{-t/\tau_3} + \frac{\gamma_2}{\tau_2} e^{-t/\tau_2}$ and the red line corresponds to the complete sum $\sum_{i=1}^{3} \frac{\gamma_i}{\tau_i} e^{-t/\tau_i}$. The Ala9 data involve a shortest and longest memory component that do not affect the subdiffusive regime in the MSD and thus are not used to predict the subdiffusive scaling. For all other proteins, all exponential memory components lie within the subdiffusive regime of the MSD and are used to compute c and d and predict the subdiffusive scaling $\alpha_{\text{sub}}^{\text{pred}}$ via Eq. (9) in the main text.

Protein	τ_m/ps	τ_D/μs	τ_1/ns	τ_2/ns	τ_3/ns	τ_4/ns	τ_5/ns	τ_{mem}/ns	$\tau_{\text{MFP}}^{\text{fol}}/\mu$s	$\tau_{\text{MFP}}^{\text{unf}}/\mu$s
λ-repressor	4.9	18.8	2.2	28	260			198	24	8
α_3D	1.5	38.7	4.9	63	4300			3760	25	24
Protein-G	3.2	15.5	2.8	43	410			343	47	33
Ala9	9×10^{-5}	0.064	7×10^{-6}	0.005	0.040	0.399	4.970	1	0.06	0.02

Table S1: Inertial time τ_m, diffusion time τ_D, and fitted memory times τ_i of the multi-exponential friction kernel $\Gamma(t) = \sum_{i=1}^{n} \frac{\gamma_i}{\tau_i} e^{-t/\tau_i}$ for different proteins. The first moment of the fitted memory kernel is given by $\tau_{\text{mem}} = \int_0^\infty t\Gamma(t)dt / \int_0^\infty \Gamma(t)dt$. The MFPTs from the unfolded state free energy minimum to the folded state minimum $\tau_{\text{MFP}}^{\text{fol}}$ and vice versa $\tau_{\text{MFP}}^{\text{unf}}$ are extracted from MD simulations.

Protein	τ_5/τ_4	τ_4/τ_3	τ_3/τ_2	τ_2/τ_1	γ_5/γ_4	γ_4/γ_3	γ_3/γ_2	γ_2/γ_1
λ-repressor			9.3	12.7			3.7	2.5
α_3D			68.3	12.8			8.2	4.4
Protein-G			9.5	15.4			5.1	5.2
Ala9	12.5	10.0	8.0	714.2	0.2	5.7	3.5	5.5

Table S2: Ratios of memory times and of friction coefficients.

Protein	γ_1	γ_2	γ_3	γ_{tot}
λ-repressor	60	148	542	750
α_3D	54	239	1960	2253
Protein-G	22	113	588	723

Table S3: Friction components γ_i of the multi-exponential fit $\Gamma(t) = \sum_{i=1}^{n} \frac{\gamma_i}{\tau_i} e^{-t/\tau_i}$ and total friction γ_{tot} for different proteins in units $\text{u}\,\text{nm}^2/\text{s}$.

	γ_1	γ_2	γ_3	γ_4	γ_5	γ_{tot}
Ala9	2.2	12	42	240	57	350

Table S4: Friction components γ_i of the multi-exponential fit $\Gamma(t) = \sum_{i=1}^{n} \frac{\gamma_i}{\tau_i} e^{-t/\tau_i}$ and total friction γ_{tot} for Ala9 in units u/ns.

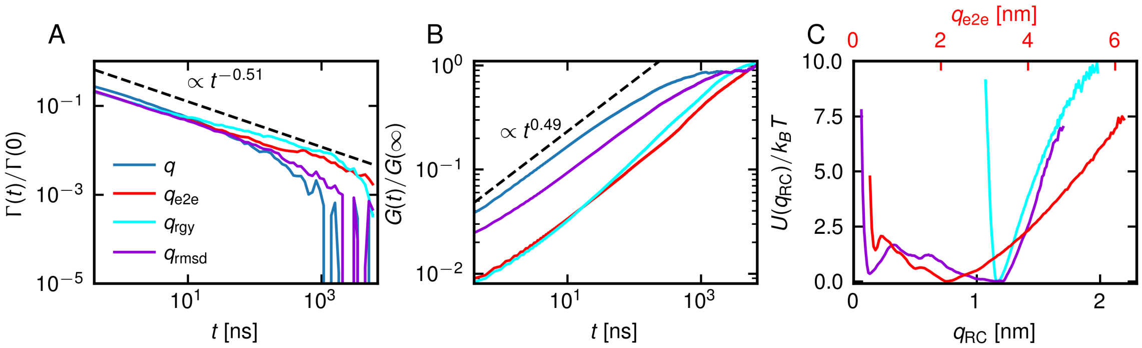

Figure S2: (A) Extracted friction kernels $\Gamma(t)$ shown for various RCs for λ-repressor (plotted for logarithmically sampled time points). (B) Integral kernels $G(t)$ corresponding to the friction kernels in (A). As a normalization, we use $G(\infty) \approx G(7000\,\text{ns})$ for easier comparison of the different RCs. The black dashed lines in (A) and (B) correspond to the power-law scaling given in Tab. 1 in the main text. (C) Free energy for the different RCs, where the x-axis of q_{e2e} is shown in red at the top and for q_{rgy} and q_{rmsd} at the bottom. The free-energy profile for the fraction of native contacts RC is shown in the main text Fig. 1C.

V Alternative reaction coordinates

End-to-end distance: The end-to-end distance for a chain of N amino acids is defined as

$$q_{\text{e2e}}(t) = |\mathbf{r}_N(t) - \mathbf{r}_1(t)|, \tag{S13}$$

where $\mathbf{r}_i$ is the position vector for location of the C_α atom in the ith amino acid residue and $i = 1, 2, ..., N$.

Radius of gyration: For a chain with center of mass $\mathbf{r}_{\text{com}}(t)$ and a set of individual particle masses m_i, the radius of gyration is given by

$$q_{\text{rgy}}(t) = \sqrt{\frac{\sum_{i=1}^{N} m_i |\mathbf{r}_i(t) - \mathbf{r}_{\text{com}}(t)|^2}{\sum_{i=1}^{N} m_i}}. \tag{S14}$$

Root-mean-squared deviation from the native state: The root-mean-squared deviation (RMSD) from the native state for the configuration of a protein at time t is given by

$$q_{\text{rmsd}}(t) = \sqrt{\frac{1}{N} \sum_{i=1}^{N} \left(\tilde{\mathbf{r}}_i(t) - \mathbf{r}_i^0 \right)^2}, \tag{S15}$$

where $\mathbf{r}_i^0$ is the position of the ith C_α atom in the native state, and $\tilde{\mathbf{r}}_i(t)$ is the position vector of the ith C_α atom at time t, for a configuration that has been uniformly translated and rotated to minimize the RMSD from the native state. The determination of the native state is described in SI section I.

Every RC describes the relaxation dynamics within the proteins and in the solvating environment differently. Therefore, the extracted friction kernels and free-energy profiles differ from RC to RC. We show the extracted $\Gamma(t)$, $G(t)$ and $U(q_{\text{RC}})$ for the end-to-end distance, the radius of gyration and the RMSD of λ-repressor in Fig. S2. As expected, the extracted friction kernels and free-energy profiles depend on the RC. Nevertheless, the friction kernels show a similar power-law behavior for the different RCs shown in Fig. S2A,B. For λ-repressor, the longest memory time of q_{e2e} and q_{rgy} is longer than for q, as indicated by the fact that the memory kernel follows a power law for a longer time, best seen in Fig. S2B. The similar power-law behavior of the friction kernels across different RCs demonstrates the robustness of our analysis of the diffusion behavior of proteins. Truncated power-law behavior might even be intrinsic to every one-dimensional RC that yields a good description of the folding dynamics. As we demonstrate in the main text, such truncated power-law behavior is well-described by a multi-exponential kernel with hierarchical order of the friction amplitudes and memory times according to Eq. (7).

VI MFPT and friction profiles from MD data

The MFPT is defined as the mean time to reach a final position q_F for the first time when starting from a position q_S. For the Langevin Eq. (10) with coordinate-dependent friction, the MFPT for $q_S < q_F$ is given by

$$\tau_{\text{MFP}}(q_S, q_F) = \beta \int_{q_S}^{q_F} \mathrm{d}q e^{\beta U(q)} \gamma(q) \int_{q_{\min}}^{q} \mathrm{d}q' e^{-\beta U(q')} \tag{S16}$$

and for $q_S > q_F$ by

$$\tau_{\text{MFP}}(q_S, q_F) = \beta \int_{q_F}^{q_S} \mathrm{d}q e^{\beta U(q)} \gamma(q) \int_{q}^{q_{\max}} \mathrm{d}q' e^{-\beta U(q')}. \tag{S17}$$

The derivative of Eqs. (S16) and (S17) with respect to q_F yields the friction profile $\gamma(q_S, q_F)$ as [4, 5]

$$\begin{aligned} \gamma_{\text{fol}}(q_S, q_F) &= k_B T \frac{e^{-\beta U(q_F)}}{Z_1} \frac{\partial \tau_{\text{MFP}}(q_S, q_F)}{\partial q_F} \text{ for } q_S < q_F, \\ \gamma_{\text{unf}}(q_S, q_F) &= -k_B T \frac{e^{-\beta U(q_F)}}{Z_2} \frac{\partial \tau_{\text{MFP}}(q_S, q_F)}{\partial q_F} \text{ for } q_S > q_F, \end{aligned} \tag{S18}$$

where $Z_1 = \int_{q_{\min}}^{q_F} \mathrm{d}q e^{-\beta U(q)}$ and $Z_2 = \int_{q_F}^{q_{\max}} \mathrm{d}q e^{-\beta U(q)}$. Here, the subscripts for folding and unfolding are reversed for q_{HB}, since folding occurs in the direction of decreasing q_{HB}. Oppositely, folding occurs in the direction of increasing the fraction of native contacts RC q. Eqs. (S18) are then used to compute coordinate-dependent friction profiles from the MD MFPT profiles, shown in Fig. S3. The resulting folding and unfolding friction profiles averaged over q_S are shown in Fig. S4.

There are two different definitions in the literature to extract MFPTs from data [6], namely the first-first passage definition and the all-first passage definition. The first-first passage time from q_S to q_F is defined as the time from the first moment q_S is reached to the first moment q_F is reached. An all-first passage time is defined as the time from any moment where the trajectory crosses the value q_S to the moment it first reaches q_F. When non-Markovian effects are present, these two definitions of first-passage events lead to different first-passage time distributions, and consequently, to different MFPT profiles [6]. In Fig. S3 we show that the mean first-first-passage time profiles and mean all-first passage time profiles are very similar for all proteins. This holds for the profiles extracted from the MD data as well as for the profiles extracted from GLE simulations. The first-first passage time is more sensitive to non-Markovian effects, which is why we choose to show the mean first-first passage profile to highlight the influence of non-Markovian effects in the main text Fig. 4 and in all figures besides Fig. S3. When we write MFPT, we thus refer to the mean first-first passage time, which we also use to extract the coordinate-dependent friction profiles.

Fig. S3 also shows that the Markovian description with the constant total friction γ_{tot} decently estimates the folding and unfolding time (minimum to minimum MFPT) for most of the proteins. However, the short- and intermediate-time dynamics, represented by the MFPTs over small distances, are only captured correctly by the GLE with the inclusion of non-Markovian effects, as similarly seen for the MSD in the main text.

The MD-simulation MFPT profiles in Fig. S5A,D for λ-repressor and Ala9 are well reproduced by GLE simulations (cyan dotted lines). For $\alpha_3 D$ and protein-G, the MD-simulation MFPT and GLE-simulation MFPT show good agreement for small distances between the start and final positions (q_S and q_F), while there are some deviations for intermediate and for long distances. In contrast, the Markovian Langevin equation with coordinate-dependent friction Eq. (10) cannot describe the MFPT profiles consistently, since neither $\bar{\gamma}_{\text{fol}}(q_F)$ alone nor $\bar{\gamma}_{\text{unf}}(q_F)$ alone, can describe both MFPT profiles, in the folding and unfolding direction. If the coordinate-dependent friction $\gamma(q_S, q_F)$ is extracted from a single MFPT folding profile with starting position q_S, it exactly reproduces the input MFPT profile by construction according to Eq. (S18); however, it does not reproduce the MFPT in the opposite direction [4]. This is shown in Fig. S6 for λ-repressor: The unfolding MD MFPT profile is perfectly reproduced by the coordinate-dependent friction profile $\gamma_{\text{unf}}(q_S = 0.75, q_F)$ that is extracted from this MFPT profile $\tau_{\text{MFP}}(q_S = 0.75, q_F)$ (by construction), whereas the folding MFPT profile $\tau_{\text{MFP}}(q_S = 0.5, q_F)$ is not.

In order to obtain a smooth coordinate-dependent friction profile, we average over several MFPT profiles with different starting points q_S, consistent with the MSD averaging over different starting points, as explained in Sec. VII. This leads to a rather close agreement of the Markovian coordinate-dependent friction prediction of the MFPT with the MD input data: dashed lines are close to folding MFPT profiles, and solid lines are close to unfolding MFPT profiles in Fig. S5. Note that for the HB_4 RC of Ala9, the folding occurs for decreasing q, whereas for the fraction of native contacts RC, folding is described by increasing q. Coordinate-dependent

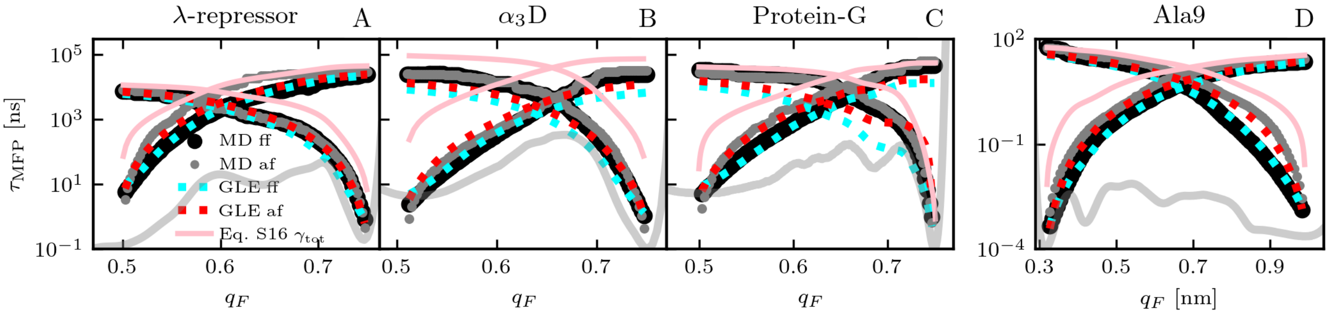

Figure S3: Comparison of mean all-first (af) passage times and first-first (ff) passage times. Passage times extracted from the MD simulations are shown as black circles for the first-first passage times and as gray circles for all-first passage times. The corresponding profiles extracted from GLE simulations are shown as red dotted lines for the all-first passage times and as cyan dotted lines for the first-first passage times. The pink lines represent the Markovian prediction of the MFPT for constant friction γ_{tot} using Eqs. (S16), (S17). Starting points for the folding profiles are: for λ-repressor, $q_S = 0.5$; for α_3D, $q_S = 0.51$; for protein-G, $q_S = 0.5$; and for Ala9, $q_S = 0.99$, nm. Starting points for the unfolding profiles are: $q_S = 0.75$ for λ-repressor, α_3D, and protein-G; and for Ala9, $q_S = 0.32$, nm. The gray lines in the background show the free energy $U(q)$.

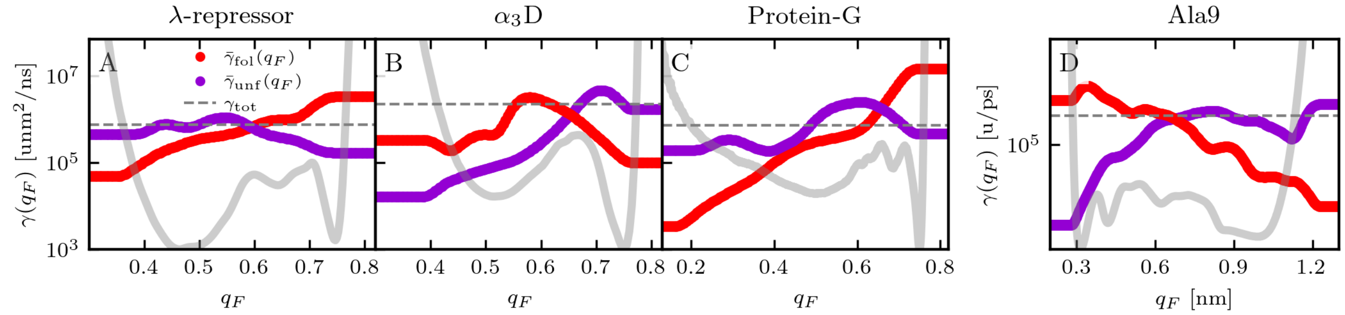

Figure S4: Average coordinate-dependent friction profiles extracted from MFPT profiles of the MD data using Eq. (S18). The profiles extracted in the folding direction $\bar{\gamma}_{\text{fol}}(q_F)$ are shown in red; profiles from the unfolding direction $\bar{\gamma}_{\text{unf}}(q_F)$ in violet. The dashed line denotes the total friction γ_{tot} from the integral over the extracted memory kernel, and the gray lines show the free energy $U(q_F)$.

friction profiles from folding MFPT profiles lead to poor agreement of the Markovian prediction with the MD data for unfolding MFPT profiles and vice versa, as shown in Fig. S5. Thus, the Markovian model with coordinate-dependent friction does not consistently describe the configurational dynamics, as also seen in the main text for the MSD. This is further underscored by the friction profiles themselves, which largely deviate when extracted from the folding or unfolding direction, as seen in Fig. S4.

VII Extracting average friction profiles from MFPT profiles

Friction profiles are extracted from MFPT profiles according to Eq. (S18) [5]. In order to decrease the noise in coordinate-dependent friction profiles, we extract MFPT profiles for different starting points q_S, as shown in Fig. S7A. This yields several $\gamma(q_S, q_F)$ curves with overlapping q intervals, as shown in Fig. S7B for the folding direction. All friction profiles in one direction, as shown in Fig. S7B, are averaged over q_S to obtain the average coordinate-dependent friction profiles $\bar{\gamma}_{\text{fol}}(q_F)$ and $\bar{\gamma}_{\text{unf}}(q_F)$ shown in Fig. S7C in the respective direction (dark green for folding and light green for unfolding). The MD simulations only rarely sample positions of high free energy, which increases the noise of the coordinate-dependent friction in regions with high free energy, especially for high and low values of q. Certain RC values are not covered at all in the MD data because of the finite simulation length. In order to regularize the behavior of our Langevin simulations at these points, we need to define the coordinate-dependent quantities $\gamma(q)$ and $U(q)$ for all possible RC values. In Langevin simulations with coordinate-dependent friction, governed by Eq. (10), we augment the friction profiles as constant on both sides, where MD simulations cannot provide the necessary MFPT information. The free-energy profile $U(q)$ is augmented on both sides by the continuation of the cubic spline interpolation of the last available RC values.

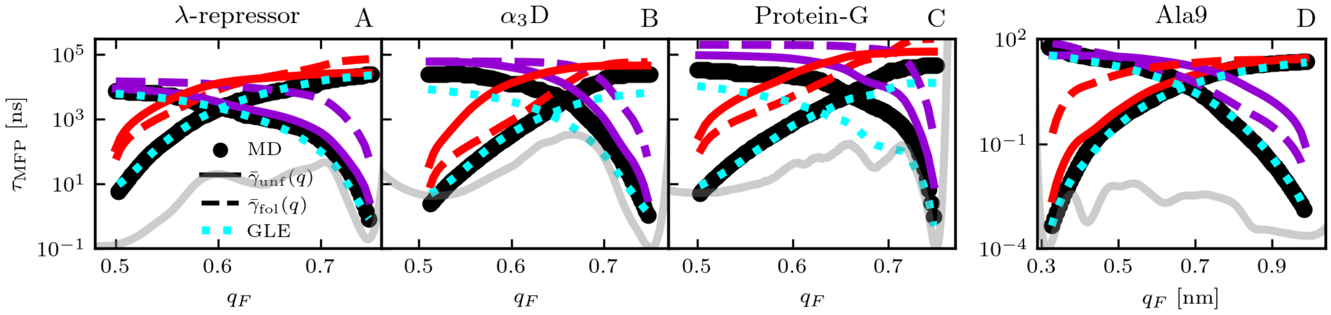

Figure S5: The MFPT extracted from MD simulations (black circles) as a function of q_F, is compared to the prediction from GLE simulation (cyan dotted lines) and the prediction from the Langevin equation with coordinate-dependent friction from folding $\bar{\gamma}_{\text{fol}}(q_F)$ (dashed lines) and unfolding $\bar{\gamma}_{\text{unf}}(q_F)$ (solid lines) according to Eq. (S16) and Eq. (S17) respectively. Starting points for the folding profiles are: for λ-repressor, $q_S = 0.5$; for α_3D, $q_S = 0.51$; for protein-G, $q_S = 0.5$; and for Ala9, $q_S = 0.99, \text{nm}$. Starting points for the unfolding profiles are: $q_S = 0.75$ for λ-repressor, α_3D, and protein-G; and for Ala9, $q_S = 0.32, \text{nm}$. The gray lines in the background show the free energy $U(q_F)$.

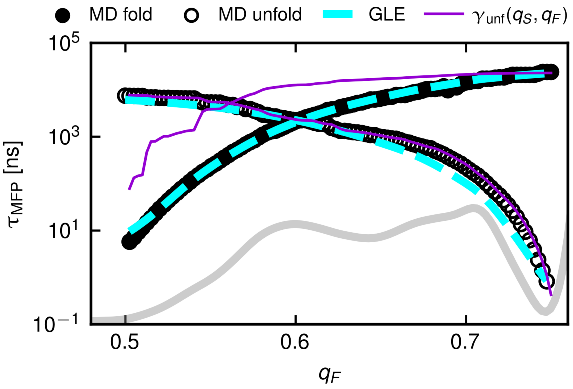

Figure S6: The MFPT profile as a function of q_F for λ-repressor, where $q_S = 0.5$ in the folding direction and $q_S = 0.75$ in the unfolding direction. The results extracted from MD simulations (black circles) are compared to the prediction from GLE simulations (cyan dotted lines) and the predictions from Eqs. (S16), (S17) using the coordinate-dependent friction $\gamma_{\text{unf}}(q_S = 0.75, q_F)$. Here, the friction profile $\gamma_{\text{unf}}(q_S = 0.75, q_F)$ for the prediction of the MFPT is extracted directly from the shown unfolding MFPT profile $\tau_{\text{MFP}}(q_S = 0.75, q_F)$ instead of using the averaging procedure explained in Sec. VII and used for the results shown in the main text. The gray lines in the background show the free energy $U(q_F)$.

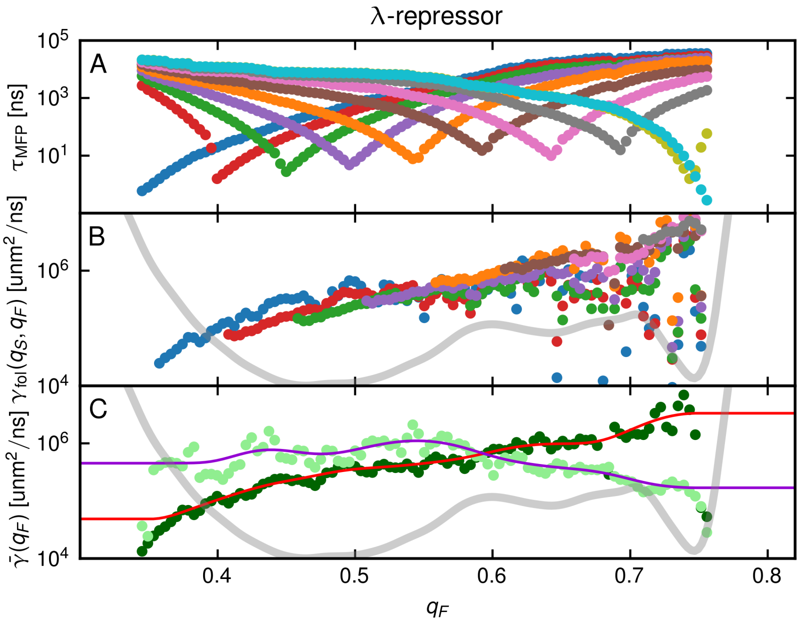

Figure S7: (A) MFPT profiles from MD simulations with different starting points q_S are shown in different colors. Starting points q_S are located at the minimum of the respective profile. The parts of the MFPT curves for which $\tau_{\rm MFP}$ increases for increasing q_F is used to compute a folding friction profile in the respective q regime. Parts where $\tau_{\rm MFP}$ increases for decreasing q_F are used to compute unfolding friction profiles in the respective q regimes. (B) All folding friction profiles calculated from the folding MFPT profiles in (A) according to Eq. (S18) are shown in the corresponding color. (C) The average over friction profiles with different starting points q_S is shown in the lower panel for the folding direction in dark green (average over profiles in (B)) and unfolding in light green. The red and violet lines represent the smoothed friction profiles that are extrapolated as constant on both sides and are also shown in Fig. S4, which are used for simulations of Eq. (10).

Finally, we smooth the friction profiles with a Gaussian window function, which yields the red and violet lines in Fig. S7C that correspond to the lines shown in Fig. S4A.

VIII Inclusion of inertial effects in addition to coordinate-dependent friction does not improve agreement with MD data

An exact expression for the MFPT is only available in the overdamped scenario described by Eq. (10). However, once the coordinate-dependent friction $\gamma(q)$ is extracted from the overdamped Eq. (S18), we can add inertial effects to the Langevin equation to test the influence of inertial effects in the presence of coordinate-dependent friction. The Langevin equation including inertial effects and coordinate-dependent friction $\gamma(q)$ reads

$$m\ddot{q}(t) = -\nabla U(q) - \gamma(q)\dot{q}(t) + \sqrt{k_B T \gamma(q)}\xi(t)\,. \tag{S19}$$

We simulate Eq. (S19) by using the same mass $m = k_B T/\langle \dot{q}^2 \rangle$ as for the GLE simulations and the friction profiles shown in Fig. S4, extracted from the overdamped theory Eq. (S18).

The MSD of simulations of Eq. (S19) (solid lines) show very similar behavior to the MSD of the overdamped version Eq. (10) (dotted lines), as seen in Fig. S8. This showcases the overdamped nature of protein folding dynamics, as demonstrated in the main text already by the fact that $\tau_m \ll \tau_D$. Therefore, the addition of inertial effects in the framework of the Langevin equation with coordinate-dependent friction still cannot reproduce the correct subdiffusive scaling exponents. Only in the long-time limit do Markovian theories capture the correct MSD behavior, in which case the dynamics are effectively described by a constant friction and the confinement of the external potential.

In Fig. S9, we compile the MSD of all models discussed in the main text. As explained in the main text, the GLE simulations agree nicely with the MD data (gray circles), and the agreement is improved by adding a

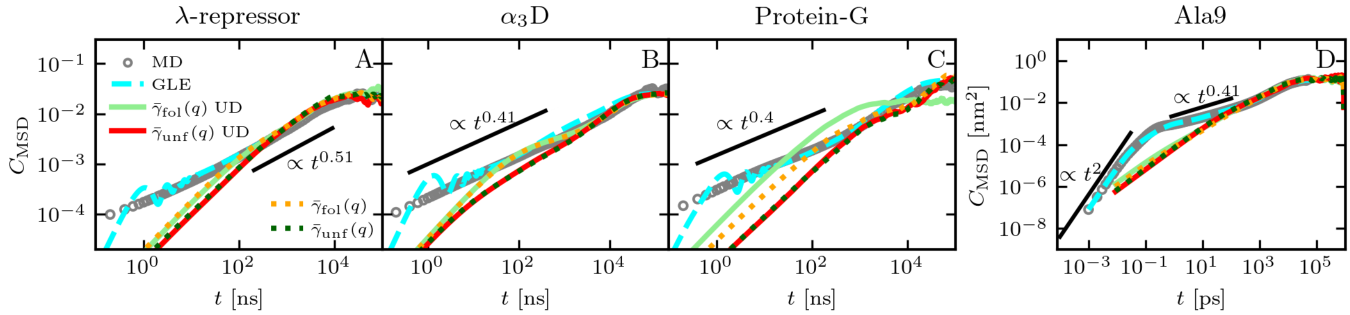

Figure S8: MSD of different proteins. Data extracted from MD simulations are shown as gray circles, where the cyan dashed lines represent the result from simulating the GLE in Eq. (1) in the main text using the respective fitted kernel $\Gamma(t)$ of Figs. 2A-D and the respective potential $U(x)$ from Figs. 1B-D. The other lines represent Markovian Langevin simulation results with the respective extracted coordinate-dependent friction profile shown in Fig. S4 in the respective potential landscape of Figs. 1B-D, where solid light green and red lines represent underdamped dynamics, including inertial effects according to Eq. (S19), and dotted dark green and orange lines represent overdamped dynamics according to Eq. (10).

friction memory component on a shorter time scale according to the mean ratios of fitted memory time scales, c, and friction amplitudes, d, in Tab. 1 of the main text. The Markovian models only capture the long-time behavior accurately and do not describe the subdiffusive scaling. The red lines show the analytical prediction [7] of the GLE with $U(q) = 0$, and the black lines represent the case for $U(q) = Kq^2/2$, with K chosen to reproduce the long-time MSD plateau value, using the variance of the position distribution $p(q)$ in Eq. (3) for the respective extracted free energy $U(q)$.

IX Addition of longer memory time contributions reduces agreement with MD data

Adding memory contributions according to the observed hierarchical pattern in the memory times and amplitudes measured in the data leads to better agreement with the MD data for the fast-folding proteins when short time scales are added, as shown in Figs. S9, S10 by the violet and red lines. In contrast, adding memory contributions on longer time scales than the largest fitted memory time, τ_3 in the case of λ-repressor, leads to worse agreement with the MSD of the MD data. The addition of a memory contribution with memory time τ_4 leads to an MSD (orange line in Fig. S10), which shows a similar deviation from the MD data as the GLE prediction using the tri-exponential fit (cyan line). However, the addition of this memory contribution increases the total friction approximately d-fold, with $d = 2.7$ for λ-repressor. This means, the addition of memory components on a longer time scale that include high friction amplitudes, leads to a total friction that is much higher than extracted from the data. Adding two contributions with memory times τ_4 and τ_5, shown by the dark-blue line in Fig. S10, leads to strong deviations from the MD MSD, especially for long times. This indicates that the longest memory time is resolved in our memory extraction technique and no longer memory times are missing.

The MSD with the additional shorter time scales in the friction kernel (violet and red lines in Fig. S10) shows reduced oscillations compared to the MSD of the other GLE simulations. There are slight deviations in the MSDs with additional shorter memory contributions on the scale of roughly 1 ns compared to the MD data. These originate most likely from an underestimation of the slope of the ballistic MSD regime from the MD data, i.e of the mean squared velocity $\langle \dot{q}^2 \rangle$, due to the low temporal resolution (see SI of [8]). In fact, the MD data show no ballistic regime in the MSD. This suggests that the time step Δ is larger than the persistence time. Therefore, our GLE model cannot capture the value of the MD MSD at the first time step, $C_{\rm MSD}(\Delta)$, for α_3D, λ-repressor and protein-G, given the time resolution of the MD data. In contrast, the short-time behavior of the MD MSD of Ala9 is perfectly captured by the GLE description, since the MD time step for Ala9 is much smaller than the crossover time from the ballistic to the diffusive regime.

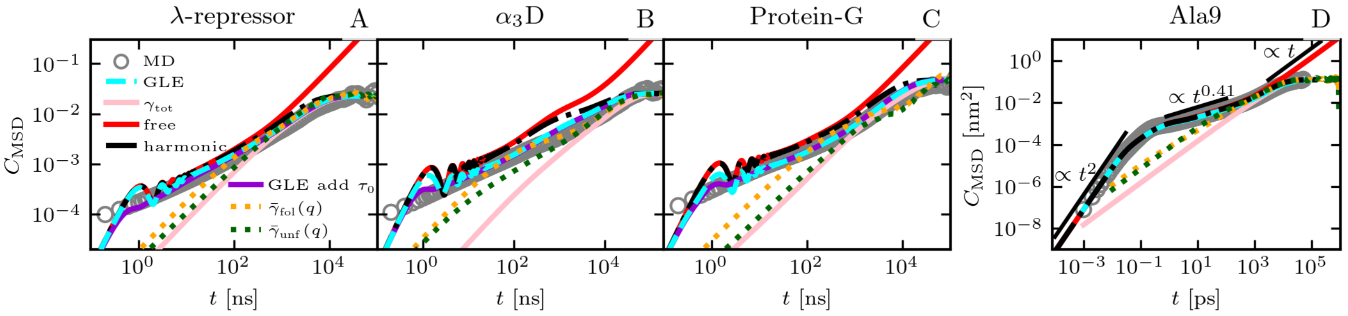

Figure S9: MSD of different proteins. Data extracted from MD simulations are shown as gray circles, where the cyan dashed lines represent the GLE simulation results using the respective fitted kernel $\Gamma(t)$ of Figs. 2A-D and the respective potential landscape from Figs. 1B-D. The violet lines result from simulating a kernel containing an additional memory component with a shorter memory time τ_0 according to Eq. (4), with values of c and d in Eq. (7) taken from Tab. 1. The red line is the analytical prediction without an external potential; the black dash-dotted line is the analytical result in a harmonic potential with the same positional variance as the free energy [7]. The dotted lines result from the simulation of Eq. (10) with average coordinate-dependent friction profiles $\bar{\gamma}_{\mathrm{fol}}(q)$ and $\bar{\gamma}_{\mathrm{unf}}(q)$ extracted from the folding MFPT profile and unfolding MFPT profile (as explained in Sec. VII) according to Eq. (S18) in the respective color. The pink lines stem from simulations of the Langevin equation in the Markovian limit using the constant total friction γ_{tot}. All simulation results are averaged over 1,000 independent trajectories, each of length 10^8 steps; further simulation details are given in Sec. IV.

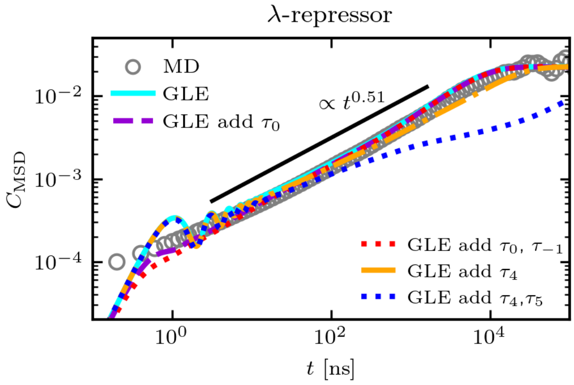

Figure S10: MSD for the fraction of native contacts from MD simulations of λ-repressor [9] as gray circles. The cyan line shows the MSD from a simulation of the GLE with the fitted memory kernel Eq. (4) shown in Fig. 2a and the free-energy profile shown in Fig. 1C. All other lines show the MSD from simulations with additional memory contributions according to the observed hierarchical pattern of memory parameters with values of c and d in Eq. (7) taken from Table 1.

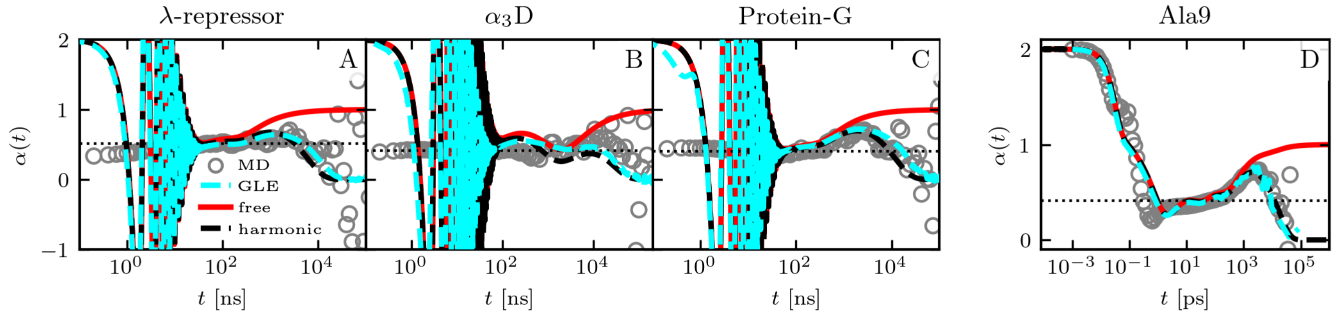

Figure S11: Comparison of $\alpha(t)$ extracted via Eq. (8) from MD data as gray circles, GLE simulations with fitted kernel $\Gamma(t)$ and extracted potential $U(q)$ as cyan lines, analytical prediction for $\Gamma(t)$ with $U(q) = 0$ in red and analytic prediction for $\Gamma(t)$ with a harmonic potential $U(q) = Kq^2/2$ in black [7]. The horizontal dotted lines show the respective value of $\alpha_{\mathrm{sub}}^{\mathrm{data}}$.

X GLE captures time-dependent exponent $\alpha(t)$

Since the GLE predicts the MSD of the proteins well, as shown in the main text, it consequently captures the time-dependent exponent $\alpha(t)$ as well, which we demonstrate in Fig. S11. Only the oscillations at short times in $\alpha(t)$ of the GLE simulations deviate from the behavior of the MD data. These oscillations are due to the missing information about shorter time scales because of the finite time resolution of the MD data and can be reduced by adding shorter memory times, as explained in the main text. The analytic predictions using the fitted friction kernel $\Gamma(t)$ with $U(q) = 0$ (red lines) describe $\alpha(t)$ well up to roughly 1 ns for Ala9 and up to 1 μs for all other proteins. The analytic prediction using $\Gamma(t)$ and a harmonic potential (black dashed line) describes the exponent $\alpha(t)$ well for all times, as it captures the long-time confinement effect leading to a plateau in the MSD and thus, a value of $\alpha = 0$ for long times. This indicates that $\alpha(t)$ is dictated mainly by friction memory effects, and the main influence of the free-energy landscape is the confinement for long times. As discussed in the main text, higher free energy barriers can introduce additional subdiffusive regimes; however, for the studied fast-folding proteins, the scaling behavior is completely governed by the memory and a harmonic confining potential. For short times, before coupling to the free-energy profile, overdamped dynamics are completely governed by memory, as explained in [7]. Since the dynamics of proteins are usually overdamped, as also shown in the main text for the studied proteins in Tab. 1, ballistic behavior occurs for even shorter times not probed in the MSD.

References

[1] Robert B Best, Gerhard Hummer, and William A Eaton. Native contacts determine protein folding mechanisms in atomistic simulations. *Proceedings of the National Academy of Sciences*, 110(44):17874–17879, 2013.

[2] Benjamin A. Dalton, Cihan Ayaz, Henrik Kiefer, Anton Klimek, Lucas Tepper, and Roland R. Netz. Fast protein folding is governed by memory-dependent friction. *Proceedings of the National Academy of Sciences*, 120(31):e2220068120, August 2023.

[3] Benjamin A Dalton and Roland R Netz. ph modulates friction memory effects in protein folding. *Physical Review Letters*, 133(18):188401, 2024.

[4] Cihan Ayaz, Lucas Tepper, Florian N. Brünig, Julian Kappler, Jan O. Daldrop, and Roland R. Netz. Non-Markovian modeling of protein folding. *Proceedings of the National Academy of Sciences*, 118(31):e2023856118, August 2021.

[5] M. Hinczewski, Y. von Hansen, J. Dzubiella, and R. R. Netz. How the diffusivity profile reduces the arbitrariness of protein folding free energies. *The Journal of Chemical Physics*, 132(24):245103, June 2010.

[6] Qingyuan Zhou, Artur Bakaev, Laura Lavacchi, Roland R Netz, and Benjamin A Dalton. Rapid state-recrossing kinetics and slow escape kinetics in non-markovian systems. *Physical Review E*, 111(6):064110, 2025.

[7] Anton Klimek, Benjamin A Dalton, and Roland R Netz. Subdiffusion from competition between multi-exponential friction memory and energy barriers. *The European Physical Journal E*, 48(8):55, 2025.

[8] Anton Klimek, Johannes CJ Heyn, Debasmita Mondal, Sophia Schwartz, Joachim O Rädler, Prerna Sharma, Stephan Block, and Roland R Netz. Intrinsic cell-to-cell variance from experimental single-cell motility data. *PRX Life*, 3:023015, 2025.

[9] Kresten Lindorff-Larsen, Stefano Piana, Ron O. Dror, and David E. Shaw. How Fast-Folding Proteins Fold. *Science*, 334(6055):517–520, October 2011.
